# Argudas: lessons for argumentation in biology based on a gene expression use case

**DOI:** 10.1186/1471-2105-13-S1-S8

**Published:** 2012-01-25

**Authors:** Kenneth McLeod, Gus Ferguson, Albert Burger

**Affiliations:** 1 School of Mathematical and Computer Sciences, Heriot-Watt University, Edinburgh, EH14 4AS, UK; 2 MRC Human Genetics Unit, Edinburgh, EH4 2XU, UK

## Abstract

**Background:**

*In situ *hybridisation gene expression information helps biologists identify where a gene is expressed. However, the databases that republish the experimental information online are often both incomplete and inconsistent. Non-monotonic reasoning can help resolve such difficulties - one such form of reasoning is computational argumentation. Essentially this involves asking a computer to debate (i.e. reason about) the validity of a particular statement. Arguments are produced for both sides - the statement is true and, the statement is false - then the most powerful argument is used. In this work the computer is asked to debate whether or not a gene is expressed in a particular mouse anatomical structure. The information generated during the debate can be passed to the biological end-user, enabling their own decision-making process.

**Results:**

This paper examines the evolution of a system, Argudas, which tests using computational argumentation in an *in situ *gene hybridisation gene expression use case. Argudas reasons using information extracted from several different online resources that publish gene expression information for the mouse. The development and evaluation of two prototypes is discussed. Throughout a number of issues shall be raised including the appropriateness of computational argumentation in biology and the challenges faced when integrating apparently similar online biological databases.

**Conclusions:**

From the work described in this paper it is clear that for argumentation to be effective in the biological domain the argumentation community need to develop further the tools and resources they provide. Additionally, the biological community must tackle the incongruity between overlapping and adjacent resources, thus facilitating the integration and modelling of biological information. Finally, this work highlights both the importance of, and difficulty in creating, a good model of the domain.

## Background

In order to gain an insight into the appropriateness of argumentation for the wider biological world this work concentrates on employing argumentation within the field of *in situ *gene expression. This section contains a short introduction to argumentation, followed by a description of the relevant previous work. Initially the focus is on *in situ *gene expression information and the areas in which argumentation may be fruitfully applied.

### Gene expression information, inconsistency, and incompleteness

Gene expression information describes whether or not a gene is expressed (active) in a location. Broadly speaking there are two types of gene expression information: one that concentrates on where the gene is expressed, and a second whose primary concern is the strength of expression. Throughout, this work focuses on the former category, in particular a technology called *in situ *hybridisation gene expression.

Information on gene expression is often given in relation to a structure - or it may be given for a point in 3D space - in a particular model organism. Here the model organism of interest is the mouse. This organism is studied from conception until adulthood. The time window is split into twenty-eight Theiler Stages. Each stage has its own anatomy, and corresponding anatomy ontology called EMAP [1]. The first twenty-six stages cover the developmental mouse: the mouse from conception until birth. Stage 27 is the new born mouse, and 28 the adult.

The result of an *in situ *experiment is represented as an image displaying a slice (2D cross section) of a mouse (from a particular Theiler Stage) in which some subsections of the mouse are highly coloured. Areas of colour indicate that the gene is expressed in that location. In addition to showing where the gene is expressed, the image provides some indication of the strength (or level) of expression. The more intense the colour, the stronger the expression.

Result images are analysed manually. A human expert determines in which structures the gene is expressed, and at what level of expression. As strength information is not the main focus of the experiment, its description is often vague using loose natural language terms such as *strong*, *moderate*, *weak *or *present*. For example, the gene *bmp4 *is strongly expressed in the future brain at Theiler Stage 15.

Once an *in situ *gene expression experiment is completed the experiment may be published in a traditional journal. Regardless of whether or not this is true, the experiment usually will be published by one (or more) online resources. Two of the main resources in the current domain of interest are EMAGE [2] and GXD [3] - both use the EMAP anatomy ontology. These online databases publish so-called *annotations *that contain particular types of information: provenance, details of the technique used, analysis of the result, and perhaps some indication of how reliable the resource believes the experiment to be. It is possible to supply information directly to the resources, and thus omit the traditional journal publication. Often such a route is favoured by large-scale projects that conduct a great number of experiments.

Although EMAGE and GXD are substantial resources they cannot be considered complete [4]. There is a range of reasons for this phenomenon including: some large scale projects publish their own results in a proprietary database, some experiments are deemed of insufficient quality by the resource curators, and others simply 'slip under the radar'. Consequently, in order to build as complete a picture of the domain as possible, it is necessary to consult multiple resources.

In addition to being incomplete, online biological resources are often inconsistent [4]. In terms of gene expression, this means that the same resource publishes one annotation suggesting the gene is expressed in a particular structure, and a second annotation suggesting it is not. As biologists treat absent and expressed as mutually exclusive, this implies an inconsistency. Due to the complexity of the underlying experiments there are a number of possible reasons for the different results, including: differences in the interpretation of results, unrecognised differences in the experiments, and human error (by either the research team or the resource's curators). As discussed previously, it is necessary to use synchronously multiple resources, but doing so raises the prospect of inconsistency between those resources, in addition to the inconsistency inside each resource.

Although there are many different methods to tackle the issues described, the use of argumentation [5] is considered in this work.

### Argumentation

*Argumentation *[5] is a multidisciplinary field that studies arguments and arguing.

An *argument *is a reason to believe something is true. This may be a formal proof, or a piece of natural language: for example, a reason to carry an umbrella. The crucial attribute of an argument is its *defeasibility*: an argument may provide a reason to believe something is true, but it does not prove it is definitely true.

Defeasibility is important because arguments can be in conflict: one argument may suggest a conclusion is true whilst a second argument intimates that the same conclusion is false. Often it is desirable to make a decision about the validity of a conclusion. As the evidence and corresponding arguments change over time, it may be necessary to revise that decision. Defeasibility allows this to happen.

Arguing (commonly called *argumentation*) is the process of using arguments to justify a point of view. This process may take place between multiple agents (human or software) inside a debate, or it may be carried out by a single agent: e.g. a political speech justifying the government's decision to increase taxes.

The subdomain of computational argumentation involves the use of computers for constructing and using arguments. There is a wide range of domains in which argumentation has been applied including: Artificial Intelligence & Law [6], ontology matching [7], medical decision support systems [8], and agent communication [9].

Argumentation in relation to biology is surprisingly rare. Jefferys *et al. *[10] use argumentation to analyse the output of a protein prediction tool. However, most work involves pedagogical efforts to improve the construction of natural language scientific arguments by students, e.g. [11].

Although argumentation is rare in biology, it is common within the medical world and is employed in a wide range of decision support systems, for example [8]. Additionally, argumentation can be used to persuade patients to change their behaviour [12], and generate pamphlets to explain complex issues to patients [13]. Although clear differences exist between medicine and biology, there are many parallels. If argumentation has been shown to work well in medicine, it should be relatively successful in similar domains, e.g. biology.

### Arguing over gene expression information

Initial attempts to tackle the twin problems of inconsistency and incompleteness are documented in length in previous work [14-16]; a brief reprise is given here.

McLeod *et al. *[14] describe an earlier system designed to tackle the above problems. In effect, this system allows a user to enquire if a gene is expressed in a particular anatomical structure from an individual Theiler Stage. Doing so causes the system to generate a number of arguments, evaluate those arguments, and present the results (argument(s) and associated evaluation) to the user.

For the arguments to be meaningful, they have to be based on expert knowledge - the expert employed for this task was the senior editor of EMAGE. Expert knowledge is captured in a series of natural language inference rules, so-called *argumentation schemes *[17]. Essentially, these schemes provide a natural language if-then (*modus ponens*) rule and an associated series of questions that can be asked to ensure the rule's application is suitable in the current context. Additionally the expert assigns a degree of confidence to each scheme.

The natural language schemes are converted into a logical form using the method described by Verheij [18]. The logic in question is a PROLOG-like logic employed by the ASPIC argumentation engine [8]. This tool provides a means to generate arguments, and conduct a virtual debate between two agents in order to determine which argument is strongest. Arguments are created by the ASPIC argumentation engine using the rules, and biological facts (information pulled dynamically at runtime from EMAGE and GXD).

The rules inherit the confidence values of their schemes, and likewise the arguments take the confidence values from their rules. When two arguments are in conflict, the engine compares the confidence values, and declares the argument with the higher value to be valid.

**Figure 1:**
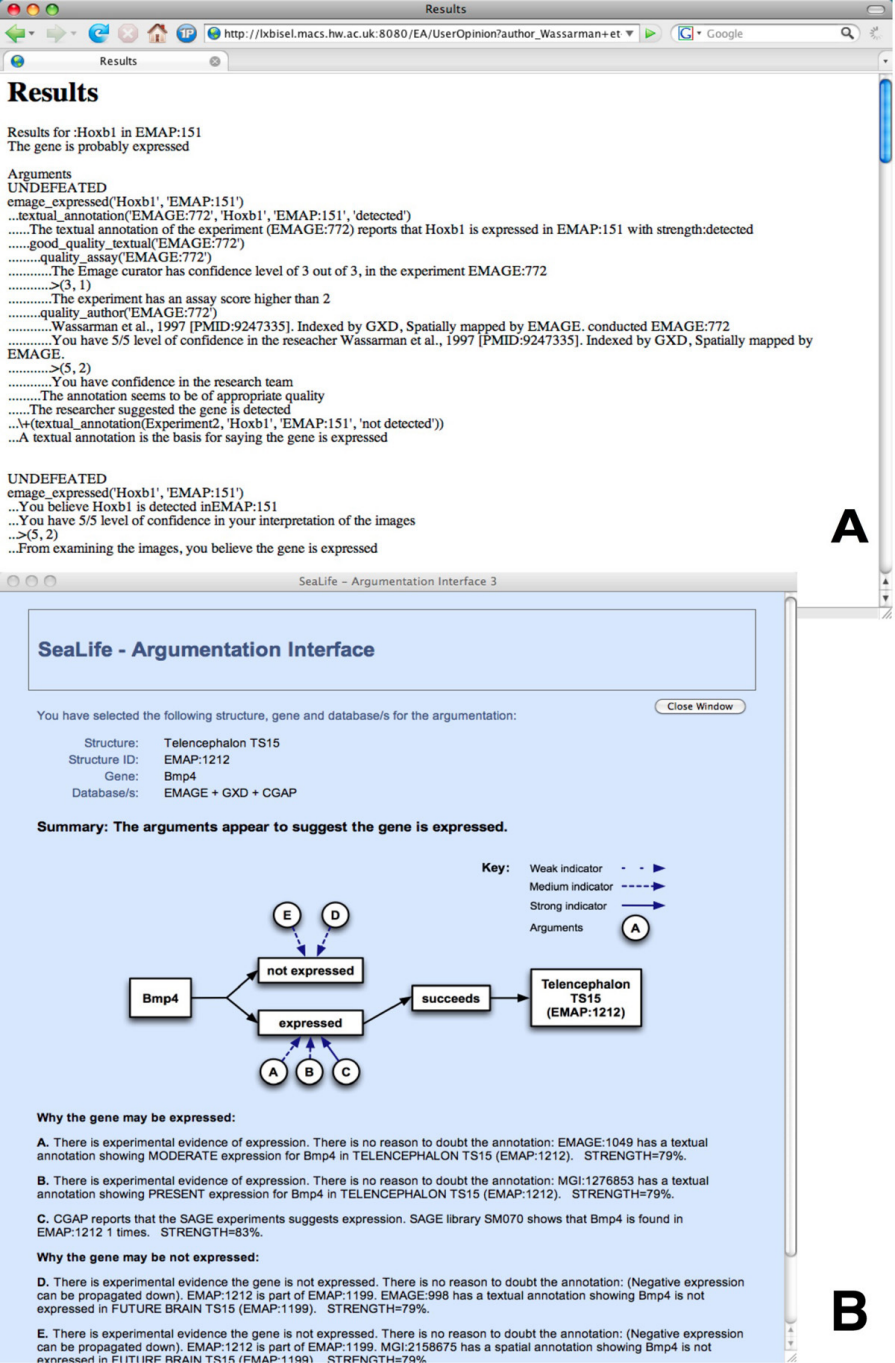
**Initial prototype graphical user interfaces**. **A**: The first prototype, results page - two true (undefeated) arguments are displayed using the argumentation engine's default presentation style. **B**: The second prototype, results page - a visual summary of the argumentation appears above a list of natural language arguments.

In McLeod *et al. *[14] the arguments are converted back into natural language and presented to the user, the presentation mechanism experts deemed most suitable. Figure 1 part **A **shows a screen shot of the results page: two arguments are displayed using one of ASPIC's in-built presentation mechanisms. Both arguments are *undefeated*, which means the system believes them to be true. Unfortunately, the default presentation style uses a mixture of natural language and logic rendering it unsuitable for use by the expected user group.

Subsequent work concentrated on the development, and evaluation of an improved interface (see Figure 1 part **B **for the updated results page). This time the arguments are presented entirely in natural language, and preceded by an image that summarises the argumentation, which is below a single line conclusion (*the gene is expressed*).

Sutherland *et al. *[16] discuss the inclusion of this work in a semantic web browser for the life sciences.

Full details of the evaluation can be found in Ferguson *et al. *[19]; though, McLeod *et al. *[15] publish the key findings. In particular, the following issues are prominent:

**subjectivity **- this affects the final conclusion (gene is/is not expressed), the schemes, and their associated degrees of belief;

**breadth of resources **- there is a need to include other gene expression resources for the developmental mouse, preferably featuring different techniques for measuring gene expression.

## Results and discussion

In 2009 work began to generate a real world tool to help tackle the issues of inconsistency and incompleteness in relation to *in situ *gene expression data for the developmental mouse - this work is undertaken as the *Argudas *project. Argudas is an evolution of the work described previously, and in [14-16]. In the former work two prototypes were developed, see Figure 1. During Argudas, the system has been refined further through the development of two additional implementations; each with a similar, yet distinct, user interface.

This section will explore the knowledge gained from the previous work, and the ways in which Argudas is informed by those examples. The discussion starts with an examination of the issue of subjectivity, then progresses to investigate the breadth of the resources included in Argudas, and concludes with a review of the issues highlighted by the first Argudas prototype.

### Tackling subjectivity

One of the problems with the previous implementations is the notion of *subjectivity*. During the evaluation it was clear that each subject had their own approach to interpreting the information contained within EMAGE and GXD. Accordingly, users disagreed with the system in three key areas: the argumentation schemes, the confidence values assigned to those schemes, and the final conclusion the system presented. Each area of conflict will now be addressed in turn.

#### Conclusions

At the top of a results page, e.g. Figure 1 part **B**, the second prototype presents a conclusion as to whether or not the gene is expressed, e.g. "The arguments appear to suggest the gene is expressed". Each time a conclusion is presented some users agree with it, yet others disagree. A plausible reason for this is discussed by Jeffreys *et al. *[10]:

Different researchers interpret data in different ways, and even the same researcher may make inconsistent interpretations, adding an unreliable and non-uniform element to data processing.

The phenomenon of subjectivity is explored, in relation to argumentation, in the philosophical writings of Perelman and Olbrechts-Tyteca [20]. Perelman and Olbrechts-Tyteca introduce the notion of an *audience *to capture the idea that each person has their own reasoning process, and thus each member of an audience will judge the same argument differently. This means that there is little point in the system trying to decide whether or not the gene is expressed. Instead the system must generate arguments for and against the gene being expressed, and allow the user to evaluate these arguments in order to reach their own decision. In effect, the system should aggregate and evaluate data, presenting the relevant data to inform the user's decision making process.

For this reason, unlike earlier work, Argudas does not decide whether or not a gene is expressed. Instead it aggregates and interprets information before summarising the material for the user.

#### Argumentation schemes

The argumentation schemes are derived from expert knowledge of how to interpret and evaluate the information in the EMAGE and GXD resources. The expert used for this task was responsible for curating the information in EMAGE, and thus interpreted and evaluated the information in these resources on a daily basis.

Unfortunately, there is little published research on the creation of argumentation schemes by a domain expert with which to compare the current work. The available literature on schemes often focuses on the dialogue and natural language aspects. The work of Silva *et. al*. [21] is interesting because it starts with already documented "reasoning templates" (effectively diagrammatic argumentation schemes) and asks a domain expert to use those to explain his reasoning, customising them if necessary, during case-based reasoning. The process of customising the schemes provides a mechanism to help the expert describe his knowledge. However, no such pre-existing schemes exist for the current domain.

Shipman and Marshall [22] discuss the problems of working with a number of knowledge formalisms, including argumentation. Although their work does not focus directly on the application of schemes, a number of their ideas do transfer over, for example:

Tacit knowledge is knowledge users employ without being conscious of its use [23]. Tacit knowledge poses a particularly challenging problem for adding formal structure and content to any system since, by its very nature, people do not explicitly acknowledge tacit knowledge.

The notion that experts are not aware of all their own knowledge presents a massive impediment for all knowledge-based approaches. Similar ideas can be found in the work of Bliss [24], which suggests that experts develop *mental models *of concepts and processes that can be very hard for them to access. Such knowledge, referred to as *deep knowledge*, is unarticulated. Knowledge that has never been articulated can be extremely difficult for the expert to recall [24-26]. The biological expert, used in this work, was being asked to provide tacit knowledge, and struggled to do so.

#### Degrees of confidence for argumentation schemes

In order for the argumentation engine to argue, it requires not only a series of logic rules (derived from the schemes) but additionally that each rule has an associated confidence score. These scores, so-called *degrees of belief*, should record the expert's confidence, in each rule, in such a way that rules with a high value produce better arguments than rules with a low value. The argumentation engine uses the scores to settle conflict, decreeing that when two arguments oppose one another, the argument with the higher value wins.

During the evaluation of the second prototype the biological expert disagreed with his own assignments, which raises questions over the reliability of their capture. However, this is not the only plausible explanation, Bliss [24] suggests that mental models naturally evolve as the person gains experience and knowledge, or as a result of the person consciously thinking about their processes and knowledge. Consequently, the act of asking an expert to document their knowledge can change that knowledge, and thus render the assigned confidence value redundant.

A further issue to be aware of when dealing with experts, is the reliability of the individuals themselves. As Walton [27] notes, the users of expert opinion often are not capable of judging the quality of that opinion and thus simply apply it:

It is quite common for presumptions to be based on expert opinion where the person who acts on the presumption - not being an expert - is not in a position to verify the proposition by basing it on hard evidence within the field of expertise in question.

Walton [27] goes on to caution that such experts, or authorities, may not prove to be reliable:

But even when they are nonfallacious, as used in a dialogue, appeals to authority are generally weak, tentative, presumptive, subjective, and testimony-based arguments. They are inherently subject to critical questioning, or even rebuttal, on various grounds - especially on grounds relating to the reliability of the source cited.

Walton's opinion is backed by a number of scientific studies, as Hansson [28] reports:

Experimental studies indicate that there are only a few types of predictions that experts perform in a well-calibrated manner. Thus, professional weather forecasters and horse-race bookmakers make well-calibrated probability estimates in their respective fields of expertise [29,30]. In contrast, most other types of prediction that have been studied are subject to substantial overconfidence. Physicians assign too high probability values to the correctness of their own diagnoses [31]. Geotechnical engineers were overconfident in their estimates of the strength of a clay foundation [32].

These quotes combine to illustrate the difficulty in using expert opinion - often it is not correct, and the user has no way of knowing what (s)he can trust. As such, it is conceivable that the expert did not assign the correct confidence values. Furthermore, the authors were incapable of verifying the quality of the assignments.

#### Argudas' solution

The previous work demonstrates a need for an improved set of schemes and confidence values. Two significant barriers appear to exist: the difficulty in the expert articulating his own knowledge, and the reliability of an expert's opinion. Instinctively, the solution to both of these issues is the inclusion of further experts in the process. Having a second expert allows ideas to be communicated and evaluated by people in different ways. Thus helping to reduce both the subjectivity and the workload on an individual expert making it easier for them to generate the required output.

Regrettably, Argudas did not have the resources to restart the full scheme generation process. Consequently, as described later, Argudas employed two experts to review the schemes and assign new confidence values. These amended schemes and values are used in Argudas' first prototype (3^*rd *^overall).

Clearly having two experts means the possibility of two different points of view. Hence, when they both agree, the probability of the degree of confidence being accurate increases. Disagreement is beneficial too, because through it new insights are discovered. It seems obvious that if the schemes had been produced by multiple experts the range and diversity of the schemes would have been broader. Furthermore, if two experts have to agree the natural language used to document the schemes, the number of ambiguous phrases should be reduced.

Yet working with multiple experts can cause a number of difficulties. Expert biologists are often geographically disparate. This in conjunction with their workload means it may be difficult to bring the experts together. Furthermore, there is an obvious requirement for a formal resolution process to help dissect and settle differences of opinion. Finally, it must be acknowledged that not all disagreements can be rectified, and that a mechanism for incorporating differences of opinion must exist. These issues point to the requirement for a framework that enables biologists to work together in order to generate the schemes. Lindgren [33] is developing such a framework for the use case of dementia care; however, it is still at an early stage, and cannot be employed here.

### Extending Argudas for richer argumentation

Argudas aims to improve on previous work with the integration of further resources - more resources means extra information and potentially richer arguments. Initially the microarray data contained in the ArrayExpress [34] resource was targeted. This highlights a number of integration issues that have yet to be resolved.

Firstly, the ArrayExpress resource does not use the EMAP anatomy ontology. Secondly, accessing the data held by ArrayExpress was difficult as they did not provide a direct programmatic access to their database. Instead access was via a RESTFUL web service, which provided limited functionality and did not allow access to the data required by this work. Initially it was impossible to ask for all the genes expressed in a healthy mouse's pancreas at stage 24 because ArrayExpress did not compute multi-factor statistics. That is, they computed which genes were expressed in the pancreas and which genes were expressed in stage 24 separately and there was no way of presenting the intersection at that time. The team behind the resource were working on improving this interface and claimed that such functionality would become available in the future; however, the delay was problematic because of the time constraints associated with Argudas. Finally, ArrayExpress had less data for the developmental mouse than expected: only three stages were covered. Comparing the costs and benefits it was decided not to pursue this integration further.

As work on ArrayExpress stopped an investigation of the Allen Brain Atlas (ABA) [35] and Gene Expression Nervous System Atlas (GENSAT) [36] began. Both of these resources are databases of in situ experiments focusing predominately on the adult mouse's nervous system, i.e. brain. The latter project makes available a full database dump. The former supplies an extensive range of RESTFUL interfaces that provide access to the desired information.

However, bringing the data from these two new resources into Argudas is problematic. Neither resource uses the EMAP anatomy - as these resources focus on the brain they have a far finer granularity for the brain structures than EMAP. Hence it is necessary to attempt some form of mapping from their respective anatomies to EMAP. Secondly, these resources use their own measures to describe the level of expression, GENSAT natural language terms and ABA floating point numbers, which also must be mapped across to the corresponding EMAGE/GXD terminology. EMAGE and GXD use very similar labels, for example when a gene is not expressed in a particular structure GXD describe the gene as *absent *whereas EMAGE use the term *not detected*.

Mapping between the different anatomy ontologies employed by the resources is based on a series of alignments produced by Jiménez-Lozano *et al. *[37]. As both GENSAT and ABA have a finer granularity than EMAP, mapping from those resources to EMAGE/GXD commonly results in a loss of precision.

The second task is straightforward for GENSAT as their choice of labels is similar to EMAGE's, and in turn GXD's. Whereas EMAGE has *not detected*, *detected*, *weak*, *moderate*, and *strong *GENSAT has *not done*, *undetectable*, *weak signal*, and *moderate to strong signal*.

Mapping EMAGE/GXD expression levels to ABA is a more complex task. There are three different measures of expression level published by ABA. Firstly there is the raw experimental information, secondly there is the average information (across all the experiments for a particular gene and structure), and finally there is a mathematical aggregation of the expression level and expression density. For current purposes, the first class of information is most suitable. Subsequently, the ABA generated expression level mappings must be applied. These mappings are a series of cut-offs that determine whether the expression level is *not expressed*, *weak*, *moderate *or *strong*. There are different limits for different parts of the brain. In order for the limits to be applied to the structures lower down in the anatomy hierarchy, the limits need to be propagated through the brain in a similar manner to the gene expression information.

When this work has been done it is necessary to determine what level of integration is appropriate for these resources. At the most basic level it would be possible to merely report the results contained in ABA and GENSAT. If either of these resources agreed with an annotation from EMAGE/GXD, it would increase the confidence in that annotation. Fully integrating ABA and GENSAT would require the generation of schemes for these resources, which is substantially more work and would necessitate involvement from a resource expert. In the case of ABA such an approach may not be fruitful; ABA does not publish all the data it collects, accordingly some of the attributes provided by an expert may be hidden from the public, and thereby Argudas. The restricted resources of Argudas meant that only the former option was realistic.

Although an interested biologist may raise a number of concerns regarding the anatomy and expression level mappings described above, currently there is no better way of aggregating data between the four resources of interest.

### Further issues identified during the development of Argudas

The first Argudas prototype tests using the revised schemes and confidence values. For the reasons discussed before, it removes the final conclusion and instead presents a list of textual arguments. When this initial system was demonstrated to a group of end users they raised concerns over the number of arguments shown and the style of their presentation. These topics shall be explored in greater depth before a possible conclusion is presented.

#### Too many arguments

During Argudas' development it became evident that the number of arguments generated varies enormously. For some queries there are no annotations and therefore no arguments. With other queries over ten annotations are retrieved from EMAGE and GXD, accordingly a large number of arguments are produced: arguing for *bmp4 *- future brain in stage 15 generates two hundred and fifteen arguments. Clearly, no biologist will read all the arguments, hence there can be no guarantee that (s)he will read all the important information. This realisation led to the conclusion that the potential number of arguments is too high, and steps were taken to reduce it.

Although all of the arguments are unique in terms of their content (wording, and order of words) semantically several arguments seem to duplicate one another. Identifying semantically *equivalent *arguments is not a minor task. The definition of equivalent seems to depend on the individual using the system and the biological task they wish to perform.

There are a number of common interpretations and actions that are not appropriate for certain biological tasks, and which individual biologists may, in general, reject. For example, the EMAP anatomy ontology is defined using part-of relationships. Consequently, positive levels of expression are routinely propagated up the ontology to higher level structures; for instance, if *bmp4 *is weakly expressed in the telencephalon, it is normally correct to say that *bmp4 *is weakly expressed in the future brain. Nevertheless, many biologists prefer direct annotations over propagated ones, thus if a second annotation suggests *bmp4 *is not detected in the future brain, the second annotation would take precedence.

Likewise, there is a similar problem with the granularity of information desired. Finding two distinct annotations with the same conclusion is a powerful argument for trusting the conclusion. However, the granularity of information desired affects the decision as to whether or not the annotations are in agreement. Imagine there are two annotations: one annotation suggests *bmp4 *is strongly expressed in the future brain, and a second annotation demonstrates *bmp4 *is weakly expressed in the future brain. If the biologist is attempting to determine if the gene is expressed or not expressed, then these annotations may be taken to agree. Yet, if the aim is to determine the level of expression, these annotations are conflicting.

The goal of reducing the number of possible arguments is further hindered by a request for more positive aspects to be highlighted. For instance, although an argument is created when the probe (used to detect the presence of an expressed gene) information is absent for an experiment, no argument is created when it is present.

In summary, Argudas' users appear to wish for a broader range of prospective arguments, and yet a smaller number of realised arguments. Reconciling these competing aims seemed improbable, until someone remarked that the problem was not the volume of the arguments but the amount of text to be read. It transpired that a significant number of potential users wanted to scan information rather than read it.

#### The notion of argument reconsidered

Previous work, and the initial version of Argudas, use the ASPIC argumentation engine to generate and evaluate arguments inside a virtual debate. These arguments are presented to the user as a natural language paragraph - this display mechanism is chosen as it is the preference of the original expert. However, feedback suggests this choice is subjective [15]. Furthermore, the potential for a large number of arguments to be generated appears to imply that the original approach is sub-optimal. There is a clear need to find an alternative method for displaying arguments.

During internal discussions it was proposed that the argumentation mechanism should be reconsidered. This approach was based on the belief that users wanted quick access to certain key attributes of the annotation. Theoretically there is no need to employ the argumentation engine to create and evaluate arguments. Instead, the most important schemes (as identified by the scheme revision process), should be the basis for a range of key attributes that describe the annotation. The schemes indicate whether or not the information stored in EMAGE/GXD for a particular annotation should increase or decrease a user's confidence in that annotation. As such, Argudas should extract information from the resources and present it, with associated key highlights, to the user. It then becomes the duty of the user to evaluate the information.

**Figure 2:**
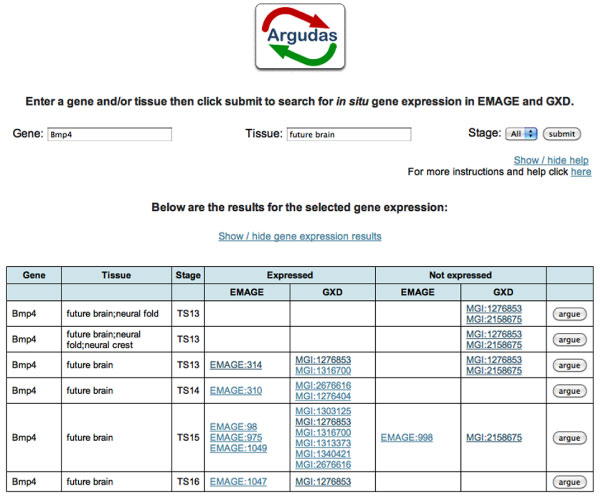
**Argudas prototype interface; part one**. Mock-up of the user interface: simple form allows user to search for gene and/or anatomical structure in relation to a particular Theiler Stage. Doing so produces a table summarising the relevant annotations found in EMAGE and GXD.

In order to test this hypothesis two mock interfaces were created and evaluated. There are three steps to each interface. The first two steps are the same: select a gene and/or structure of interest; report on the available annotations and allow the user to ask for more information if desired. Figure 2 shows both of these: initially the query is *bmp4 *- future brain in all stages; the query causes all combinations of the gene and structure to be displayed in a table. The table presents all relevant annotations, summarises what each annotation shows, and provides a link to the resource's web page for that annotation.

In some situations the table in Figure 2 will be enough to resolve a biologist's question; i.e. it is clear that *bmp4 *is expressed in the future brain in stage 14. On the occasions when the table is not helpful, or does not provide enough information, clicking the *argue *button provides a range of arguments.

**Figure 3:**
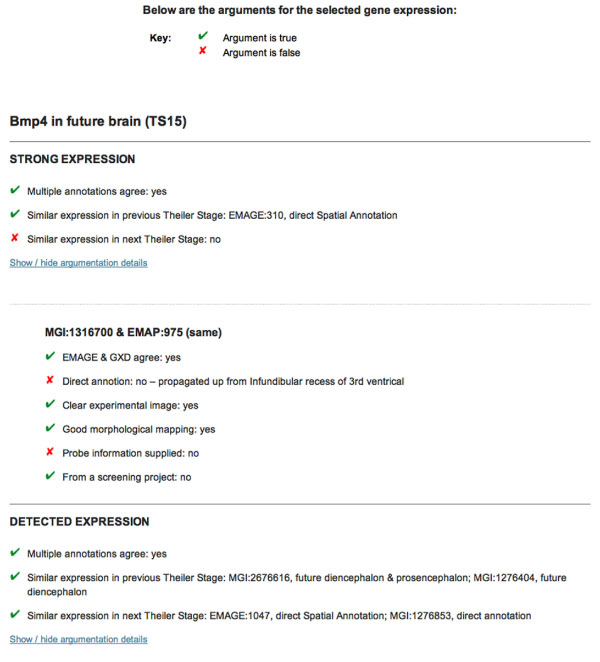
**Argudas prototype interface; part two**. Mock-up of the user interface: potential display mechanism for arguments. Clicking on an *argue* button from Figure 2 produces an output where different attributes are assigned a positive (tick) or negative (cross) indicator. Positive indicators demonstrate the annotation is more likely to be correct.

In the first mock interface a number of textual arguments are displayed - in a similar manner to Figure 1 part **B**. The second interface can be seen in Figure 3 - the 'arguments' are now a list of key attributes such as *multiple annotations agree*. Whether or not an attribute should strengthen a user's confidence in the annotation is indicated with a tick or cross. The attributes are divided into two layers - firstly by expression level, and then by annotation. For each level of expression there are three attributes that indicate how likely that level of expression is. Asking for more information causes the second layer of attributes to appear. This allows the user to evaluate the annotations individually, and collectively as a group that promotes a specific expression level.

#### Argument presentation reconsidered

The mock interfaces, shown in Figures 2 and 3, were explored with the help of two expert users. During this small informal exercise (described later in the Methods section), the test users were kept apart; however, they reached identical conclusions:

1. the revised interface, using key attributes rather than textual arguments, is an improvement;

2. a further improvement could be made by placing the content of Figure 3 into a table.

**Figure 4:**
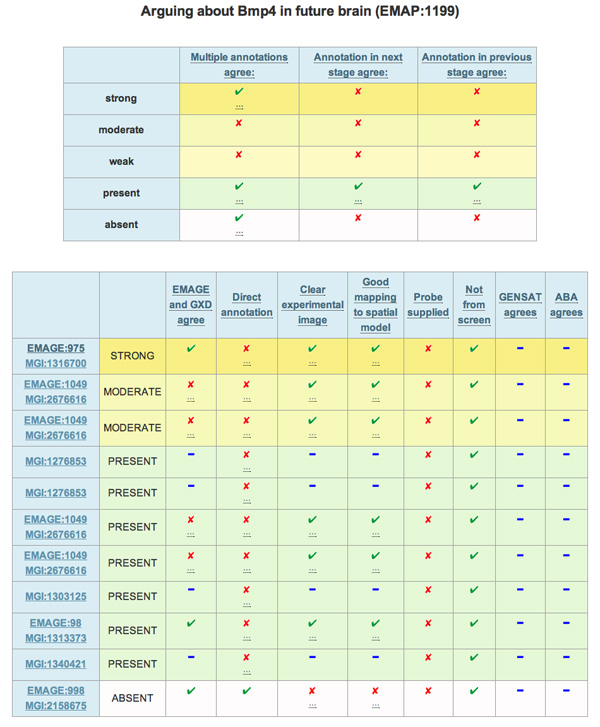
**Argudas final interface; presentation of arguments**. Implemented display mechanism for arguments. Important data attributes are columns in the tables. The two tables in this Figure are beneath a table similar to that shown in Figure 2. The top table, in this figure, features strength of expression as the rows. A tick indicates a positive reason to trust the level of expression, a cross provides a reason to doubt the level. The three dots underneath a tick or cross indicate that more information is available as a mouse-over, e.g., the tick for 'strong - multiple annotations agree' presents the experiments with matching annotations. The final table has one row dedicated to each annotation. The strength of expression is colour co-ordinated with the first table. The key is the same, with the addition of blue dashes reporting that data is unavailable.

Separately, the experts both had the idea contained in Figure 4. Effectively, the important schemes become columns, with individual annotations represented as rows. Ticks indicate positive aspects that suggest the annotation is more likely to be correct, with crosses having the opposite semantics. Blue dashes inform the user that a piece of information is unavailable.

Enacting these recommendations has a major side effect: there is no need to perform computational argumentation. As such, there is no need for the argumentation engine. Instead the user now performs the argumentation themselves using the aggregated and curated information presented by Argudas.

This change helps address the issue of subjectivity, because the user is now able to apply his/her own confidence values and criteria to the decision making process. Furthermore, because the user is able to build his/her own arguments, Argudas becomes a more flexible tool. For example, it is now possible to use Argudas to decide where, and at what level, a gene is expressed.

Implementing these changes results in the second, and current Argudas system (the user interface can be seen in Figure 4). One addition to the advice offered by the expert users is the inclusion of mouse-overs to present extra information. Discrete dots underneath a tick/cross, or column title, indicate that more information is available if the user hovers his/her mouse over the dots. For example, hovering the mouse over the dots in the cell for 'strong - multiple annotations agree' (Figure 4, top table) reveals the experiment(s) that suggest the gene is strongly expressed.

The evaluation of this system, described later, demonstrates that the changes are both appropriate and effective because they allow users to quickly scan the important information.

### Future work

This work set out to model expert knowledge and use it to reason with information sources available through the Internet. In the current use case, this appears to be beyond the scope of what a typical end user wishes. However, the current use case is relatively constrained, and thus contained: it focuses on one kind of gene expression information for one model organism. Extending the use case to include different types of biological information, for example gene regulatory networks, makes the use case considerably more complex. As the intricacy of the biological investigation increases, the need for user support likewise increases. Argumentation is one possible support mechanism.

Another avenue for future work relates to CUBIST, Combing and Uniting Business Intelligence with Semantic Technologies, an EU FP7 project that aims to combine the essential features of Semantic Technologies, Business Intelligence, and Visual Analytics. Data from both unstructured and structured sources will be federated within a Business Intelligence enabled triplet store, before visual analysis techniques such as Formal Concept Analysis [38] are applied. One of the project's three use cases involves the gene expression data described in this paper. Although it is early in the life of the CUBIST project, a semantic Extract Transform Load [39] process that includes computational argumentation may be envisioned. The inclusion of argumentation may provide an intelligent transformation of data, and a user-friendly explanation of the transformation.

## Conclusions

This paper describes the rationale for generating an argumentation tool (Argudas) to tackle the inconsistency and incompleteness found in *in situ *hybridisation gene expression resources for the developmental mouse. Furthermore, it discusses the development of Argudas highlighting some of the critical problems still outstanding.

Although the paradigm of argumentation initially seemed promising for this use case, it is clear that in this case, biologists are not interested in the full potential of argumentation. To them the ability to generate and automatically evaluate many arguments is not of primary interest. Nor is the presentation of a conclusion - they wish to make that decision. As such there is no place in Argudas for the version of computational argumentation carried out in previous work. Instead the concept of an argument, as *a reason to believe something*, can be used to present key attributes that provide a good indication of whether or not an annotation can be trusted, and thus whether or not a gene is expressed.

Computational argumentation may not be wholly appropriate for the current use case, yet that does not mean that the technology cannot be applied to other domains within the Life Sciences. The current use case is restricted in terms of its complexity. For more elaborate situations, in which data from multiple fields is aggregated for knowledge generation, the user support provided by argumentation may be more valuable.

For argumentation to be successfully applied within the biological domain, this work shows that two strands of activity are important. Starting within the biological domain, it is clear that integration of resources is essential. To tackle the incompleteness of a single biological resource it is necessary to aggregate data from other sources. Argudas tried to expand beyond its initial online sources; yet, doing so introduced a number of challenges. All the resources featured in this paper are essentially conducting the same task for the same type of information; however, the resources use different anatomy ontologies, terminologies and methods. Accordingly, integrating data is not a straightforward task.

The issues faced when integrating data across sources are not unique to *in situ *gene expression for the developmental mouse. They are applicable to all domains which involve experimentation on model organisms. Currently, there is no adequate solution to these difficulties and users have to accept some limitations.

The second requirement, for argumentation to be successfully utilised, is an improvement in the quality and availability of argumentation methods and tools. The argumentation community must tackle the open question of how best to present arguments and argumentation, and produce a collaborative mechanism to enable biologists to model and record domain knowledge themselves. Finally, there is a requisite for the community to have their work freely available through the creation of open, reliable, and scalable argumentation toolkits. Currently, there are few such resources available.

Even with these issues resolved, the effectiveness of computational argumentation in biology hinges on the quality of the domain modelling. Regardless of the application domain, the effort required to model domain information is significant. This cost presents a substantial barrier to the successful adoption of computational argumentation within biology, and raises questions over whether argumentation can reach its full potential within this domain. Yet the same is true for the Semantic Web, and as James Hendler and others [40] have stated - a little semantics goes a long way.

Whilst it must be acknowledged that the tools to support argumentation are currently too immature for real world use, and the principles behind those tools require refinement, it seems clear that the concept of argumentation has real potential within the life sciences.

## Methods

In this section a number of activities relating to the work described previously will be documented. The procedure for accessing and revising the argumentation schemes and their confidence values will be tackled first. Subsequently the informal evaluation of the first Argudas prototype will be recounted, before the evaluation of the second prototype is considered. The implementation of Argudas will not be discussed as that is largely a software engineering exercise.

### Revising the argumentation schemes and confidence assignments with multiple experts

As discussed above, when the previous work was evaluated [15] a number of evaluation subjects disagreed with the expert's schemes and his assignment of degrees of confidence to those schemes. Argudas did not have the resources to create a new set of schemes as this was a sizeable task; nevertheless, it was possible to review the schemes and their confidence values. To this end, two experts were asked to appraise the list of previously generated schemes. Separately, each expert awarded each scheme a score:

**0 **disagree with the scheme, i.e. reject;

**?** don't know - scheme is very weak and is on the border between being rejected and being classified as a weak scheme;

**1 **weak scheme, i.e. low confidence;

**2 **moderate scheme, i.e. medium confidence;

**3 **good scheme, i.e. high confidence.

In total, the two experts were asked to assign a score to sixty-eight schemes. The experts completely agreed upon - that is they gave exactly the same score to - sixteen schemes. A further thirty-three schemes were assigned a similar score. The notion of *similar *being defined as an adjacent score, i.e. if one expert assigned **2**, then either a **1 **or a **3 **would be classified as similar. If the two experts assigned scores that were neither adjacent nor exact matches, they were deemed to disagree - this happened with nineteen schemes.

In conclusion, the experts broadly agreed on 72% of the schemes. This left 28% of the schemes for which the disagreement was marked. Regrettably, one of the experts emigrated shortly after this exercise was completed and was no longer available to assist in the development of Argudas. Therefore this disagreement was never resolved, nor was its root cause investigated.

Potentially the source of the disagreement was very interesting, as it was not clear whether the conflict between the experts was caused by a genuine difference of opinion or a difference of interpretation. As the schemes were written in natural language, the latter is a distinct possibility.

### Reconsidering the presentation of arguments

Originally arguments were presented entirely in natural language, e.g. Figure 1 part **B**. An alternative was to summarise the information by displaying only key attributes, e.g. Figure 3. This small informal exercise attempted to decide which mechanism was more appropriate for the potential users.

The two presentation styles of argument were evaluated with the assistance of two expert users from the Medical Research Council's Human Genetic Unit [41]. One expert had participated in the previous revision of argumentation schemes and confidence values. The second expert had attended a presentation on Argudas, but had not previously had a role in Argudas' development. The expert evaluations were undertaken independently with no discussion between the experts prior to the evaluation.

Each expert user was presented with a description of the planned evaluation, then a structured walkthrough was conducted. Using a protocol, the user was guided through each interface using the same search example: *bmp4 *- future brain - stage 15. They were asked to raise any issues or aspects they liked or disliked while undertaking the interface evaluations. The experts were then asked to score the interfaces out of ten in terms of their usability. Ultimately, a limited set of questions was asked to determine the user's opinions on the requirements for refining aspects of the interface and argument presentations.

In particular, one question directly asked which style of presentation the subject preferred. A second question asked how their favoured approach could be improved further. As discussed above, the users provided very similar answers, with a clear preference for displaying only key attributes. Additionally, both test subjects proffered the idea of using tabulation to improve the presentation of the attributes.

### Evaluation of second Argudas implementation

An evaluation of the new interface and latest version of Argudas, represented by Figure 4, was undertaken with eight users (consisting of biologists, bioinformaticians, and computational biologists) working at the Medical Research Council's Human Genetics Unit. Work pressures necessitated the restriction of each evaluation session to twenty minutes.

The evaluation comprised a structured walkthrough with the user being guided through a scenario of a typical Argudas query. This was followed by a usability evaluation questionnaire and a number of open-ended questions to gather the user's opinions on the presentation of results. Due to the small number of users, results were largely qualitative rather than quantitative.

Users were asked to rate their opinion of a number of aspects of the Argudas system using a five point scale. Considering the response time of the system: seven users rated it as "excellent" and one as "good". Focusing on the appearance of Argudas: one user described it as "excellent" and seven as "good".

The user ratings for how easy the three results tables were to understand favoured "easy" and neutral, with only two users finding the second and third results tables (e.g. Figure 4) "difficult" to understand.

Whilst conducting the evaluation, the observer noted that five of the users appeared to not see the key explaining the tables, suggesting that the key should be placed in a position on the page that makes it more prominent.

Failure to spot the key meant that the same users did not read the explanation of the mouse-overs that it contained. This was believed to be one contributing factor to the apparent inability of several users to discover the mouse-overs. Another relevant factor may be the deliberately discrete nature of the underlining used to indicate the presence of a mouse-over. Attempts to ensure the underlining did not intrude too significantly, appear to have made the visual clue too difficult to see.

A number of general conclusions were drawn from user comments made during the evaluation. For example, the idea of triggering help information by mouse-over. Additionally, several users felt the results tables were too big, with the information too widely spaced. These users recommended a reduction in white space and cell size. When a user asks for more information from the top results table (e.g. Figure 2), the top table is hidden to allow more space for the presentation of the two tables from Figure 4. To see the first table again, the user must click on a hyper-link ("Show/hide gene expression results" in Figure 2). It transpires that this hyperlink should be more visible.

Overall, the conclusion drawn from the evaluation is that the evolution of the interface to a tabular display has achieved the desired aim of making the results easier to "eyeball". Accordingly, user satisfaction with the tables is largely positive. However, the mechanisms that provide the user with extra information about, and context for, the results need to be improved.

## List of abbreviations used

ABA: Allen Brain Atlas; ASPIC: Argumentation Services Platform with Integrated Components; EMAP: Edinburgh Mouse Atlas Project; EMAGE: Edinburgh Mouse Atlas of Gene Expression; EU: European Union; FP7: Framework Programme 7; GENSAT: Gene Expression Nervous System ATlas; GXD: Gene Expression Database; MRC: Medical Research Council.

## Competing interests

AB served as co-editor for this supplement, but was not involved in the review of this paper. KM and GF declare they have no competing interests.

## Authors' contributions

KM carried out the initial capture of argumentation schemes and confidence values. KM and GF jointly worked with the biological experts on the revision process. KM developed the argumentation specific, and server-side components of Argudas. GF developed the user interfaces. KM designed and carried out the Argudas evaluation exercises under the supervision of GF. Analysis of the evaluations was carried out by both GF and KM. GF wrote the section describing the second evaluation of Argudas, the remainder of the paper was written by KM. GF contributed to the overall editing of the paper, as well as image creation and manipulation. AB initiated and supervised the work before reviewing the final manuscript.

## References

[B1] Baldock R Davidson D Burger A, Baldock R, Davidson D The Edinburgh Mouse Atlas Anatomy ontologies for bioinformatics: principles and practise 2008 Springer 249 265

[B2] EMAGE Edinburgh Mouse Atlas of Gene Expression http://www.emouseatlas.org

[B3] GXD: Gene Expression Database http://www.informatics.jax.org/expression.shtml

[B4] McLeod K Burger A Guimaraes N, Isaís P Using argumentation to tackle inconsistency and incompleteness in online distributed life science resources Proceedings of IADIS International Conference on Applied Computing: 18-20 February 2007; Salamanca, Spain 2007 IADIS Press 489 492

[B5] Bench-Capon T Dunne PE Argumentation in artificial intelligence Artificial Intelligence 2007 171 619 641 10.1016/j.artint.2007.05.001

[B6] Bench-Capon T Prakken H Lodder A, Oskamp A Argumentation Information Technology & Lawyers: Advanced technology in the legal domain, from challenges to daily routine 2006 The Netherlands: Springer 61 80

[B7] Trojahn C Quaresma P Vieria R Matching law ontologies using an extended argumentation framework based on confidence degrees Law, Ontologies and the Semantic Web, Channelling the Legal Information Flood, Volume 188 of Frontiers in Artificial Intelligence and Applications, Netherlands 2008 IOS Press 133 144

[B8] Fox J Glasspool D Grecu D Modgil S South M Patkar V Argumentation-based inference and decision making - a medical perspective IEEE Intelligent Systems 2007 22 34 41

[B9] Rahwan I Ramchurn SD Jennings NR McBurney P Parsons S Sonenberg L Argument-based negotiation The Knowledge Engineering Review 2003 18 343 375 10.1017/S0269888904000098

[B10] Jefferys BR Kelly LA Sergot MJ Fox J Sternberg MJE Capturing expert knowledge with argumentation: a case study in bioinformatics Bioinformatics 2006 22 923 933 10.1093/bioinformatics/btl018 16446279

[B11] Adúriz-Bravo A Bonan L Galli LG Chion AR Meindardi E Scientific argumentation in pre-service biology teacher education Eurasia journal of mathematics, science and technology education 2005 1 76 83

[B12] Grasso F Cawsey A Jones R Dialectical argumentation to solve conflicts in advice giving: a case study in the promotion of healthy nutrition International Journal of Human-Computer Studies 2000 53 1077 1115 10.1006/ijhc.2000.0429

[B13] Green N Bickmore T, Green N, Menlo Park Representing normative arguments in genetic counseling Argumentation for consumers of healthcare, Papers from the AAAI Spring Symposium: 24 - 26 March 2008; Stanford University, USA 2006 California: AAAI Press 64 68

[B14] McLeod K Burger A Towards the use of argumentation in bioinformatics: a gene expression case study Bioinformatics 2008 24 i304 i312 10.1093/bioinformatics/btn157 18586728 PMC2718635

[B15] McLeod K Ferguson G Burger A Green N, Grasso F, Kibble R, Reed C Using argumentation to resolve conflict in biological databases Proceedings of Computational Models of Natural Argument (CMNA) 9: 13 July 2009; Pasadena, USA 2009 CMNA 15 23

[B16] Sutherland K McLeod K Ferguson G Burger A Knowledge-driven enhancements for task composition in bioinformatics BMC Bioinformatics 2009 10 Suppl 10 S12 http://www.biomedcentral.com/1471-2105/10/S10/S12 10.1186/1471-2105-10-S10-S12 19796396 PMC2755820

[B17] Walton D Reed C Macagno F Argumentation Schemes 2008 New York, NY, USA: Cambridge University Press

[B18] Verheij B Dialectical argumentation with argument schemes: an approach to legal logic Artificial Intelligence and Law 2003 11 1-2 167 195

[B19] Ferguson G McLeod K Sutherland K Burger A Sealife Evaluation 2009 Tech. Rep. 0063, Dept of Computer Science, Heriot-Watt University

[B20] Perelman C Olbrechts-Tyteca L The new rhetoric: a treatise on argumentation 1969 Notre Dame/London: University of Notre Dame Press

[B21] Silva LAL Buxton FB Campbell JA Wilson D, Sutcliffe G, Menlo Park Enhanced case-based reasoning through use of argumentation and numerical taxonomy The 20th international Florida artificial intelligence research society conference (FLAIRS-20): 7-9 May 2007; Key West Florida 2007 California: AAAI Press 423 428

[B22] Shipman FM Marshall CC Formality considered harmful: experiences, emerging themes, and directions on the use of formal representations in interactive systems Computer Supported Cooperative Work 1999 8 332 352

[B23] Polanyi M The tacit dimension 1966 Garden City, NY, USA: Double Day

[B24] Bliss J From mental models to modelling Learning with artificial worlds: computer based modelling in the curriculum table of contents 1994 Bristol, PA, USA: The Falmer Press

[B25] Schumacher R Czerwinski M Hoffman R Mental models and the aquisition of expert knowledge The psychology of expertise 1992 New York: Springer Verlag

[B26] Greca IM Moreira MA Mental models, conceptual models and modeling International Journal of Science Education 2000 22 1 11 10.1080/095006900289976

[B27] Walton DN Argumentation Schemes for Presumptive Reasoning (Studies in Argumentation Series) 1996 Mawah, NJ, USA: Lawrence Erlbaum Associates

[B28] Hansson SO Decision theory a brief introduction http://home.abe.kth.se/~soh/decisiontheory.pdf

[B29] Murphy AH Winkler RL Probability forecasting in meterology Journal of the American Statistical Association 1984 79 489 500 10.2307/2288395

[B30] Hoerl AE Fallin HK Reliability of subjective evaluations in high incentive situation Journal of the Royal Statistical Society. Series A (General) 1974 137 227 230 10.2307/2344550

[B31] Christensen-Szalanski JJ Bushyhead JB Physician's use of probabilistic information in a real clinic setting Journal of experimental psychology, human perception and performance 1981 7 928 935 6457103 10.1037//0096-1523.7.4.928

[B32] Hynes M Vanmarcke E Reliability of embankment performance predictions Proceedings of the ASCE Engineering Mechanics Division, Speciality Conference 1976 Waterloo, Canada: University of Waterloo Press

[B33] Lindgren H Winnberg P Szomszor M, Kostkova P Evaluation of a semantic web application for collaborative knowledge building in the dementia domain In proceedings of e-Health 2010 - 3rd international ICST conferencce on electronic healthcare for the 21st century: 13-15 December 2010; Casablanca, Morocco 2010 Springer 62 69

[B34] ArrayExpress http://www.ebi.ac.uk/arrayexpress/

[B35] Allen Brain Atlas http://www.brain-map.org

[B36] GENSAT: Gene Expression Nervous System Atlas http://www.gensat.org 10.1038/nn0504-483 15114362

[B37] Jiménez-Lozano N Segura J Macías J Vega J Carazo JM aGEM: an integrative system for analyzing spatial-temporal gene-expression information Bioinformatics 2009 25 2566 2572 10.1093/bioinformatics/btp422 19592395 PMC2752607

[B38] Andrews S Orphanides C Kryszkiewicz M, Obiedkov S Analysis of large data sets using formal concept lattices Proceedings of the 7th internation conference on concept lattices and their applications: 19-21 October 2007; Seville, Spain 2010 CEUR Workshop Proceedings 104 115

[B39] Kimball R Caserta J The data warehouse ETL toolkit: practical techniques for extracting, cleaning, conforming, and delivering data 2004 John Wiley and Sons

[B40] Wolstencroft K Brass A Horrocks I Lord P Sattler U Turi D Stevens R Gil Y, Motta E, Benjamins V, Musen M A Little Semantic Web Goes a Long Way in Biology The Semantic Web - ISWC 2005: 4th International Semantic Web Conference: 6-10 November 2005; Galway, Ireland, Volume 3729 2005 Berlin: Springer 786 800

[B41] Medical Research Council's Human Genetics Unit (HGU) http://hgu.mrc.ac.uk

